# Facing the Knife, Finding the Spirit? A Study of Daily Spiritual Experiences Scale (DSES) Scores in Romanian Surgical and Non-Surgical Patients

**DOI:** 10.3390/healthcare13151820

**Published:** 2025-07-25

**Authors:** Andrei Ionut Cucu, Şerban Turliuc, Mihaela Cristina Sandiuc (Pietsch), Cristina Gena Dascălu, Otilia Boişteanu, Amelian Mădălin Bobu, Claudia Florida Costea, Iulian Prutianu, Alexandru Cărăuleanu, Catalin Mihai Buzdugă, Roxana Covali, Camelia Tamaş, Adriana Hristea, Emilia Pătrăşcanu

**Affiliations:** 1 Faculty of Medicine and Biological Sciences, University Stefan cel Mare of Suceava, 720229 Suceava, Romania; andrei.cucu@usm.ro; 2 Emergency Clinical Hospital Prof. Dr. Nicolae Oblu, 700309 Iasi, Romania; claudia.costea@umfiasi.ro; 3 Faculty of Medicine, University of Medicine and Pharmacy Grigore T. Popa Iasi, 700115 Iasi, Romania; cdascalu_info@yahoo.com (C.G.D.); otilia.boisteanu@umfiasi.ro (O.B.); pruty04@gmail.com (I.P.); drcarauleanu@yahoo.com (A.C.); catalinbuzduga@gmail.com (C.M.B.); camelia6ta@yahoo.com (C.T.); patrascanu.emilia@umfiasi.ro (E.P.); 4 Medical Office of the Ștefan cel Mare National College, Social Assistance Department, Suceava City Hall, 720001 Suceava, Romania; mihaela.pietsch@yahoo.com; 5 Clinical Emergency Hospital St. Spiridon, 700111 Iasi, Romania; amelian.bobu@gmail.com; 6 Faculty of Medical Bioengineering, University of Medicine and Pharmacy Grigore T. Popa Iasi, 700115 Iasi, Romania; ana.covali@umfiasi.ro; 7 Infectious Diseases Department, Faculty of Medicine, University of Medicine and Pharmacy Carol Davila, 050474 Bucharest, Romania; adriana.hristea@umfcd.ro; 8 National Institute for Infectious Diseases Prof. Dr. Matei Bals, 021105 Bucharest, Romania; 9 Regional Institute of Oncology, 700483 Iasi, Romania

**Keywords:** spirituality, Daily Spiritual Experiences Scale, patient-centered care, surgical interventions, holistic medicine

## Abstract

**Background**: Spirituality is increasingly seen as a key component of patient-centered care, especially in serious illness or surgery. However, its role among surgical patients in Eastern Europe remains underexplored. **Objective**: To assess daily spiritual experiences among surgical patients compared to healthy individuals in the Bucovina region (northeastern Romania). **Methods**: This observational case-control study included 102 participants (51 surgical patients and 51 healthy controls), recruited between March 2023 and April 2024. Participants completed the validated Romanian version of the Daily Spiritual Experiences Scale (DSES). **Results**: Surgical patients reported significantly higher mean DSES scores (M = 66.27, SD = 16.40) than healthy individuals (M = 55.06, SD = 12.81; *p* < 0.001). Higher scores were also associated with female gender (*p* = 0.002), older age, and oncological conditions. Widowed and highly educated participants showed a trend toward higher spirituality, though it was not statistically significant. **Conclusions**: Surgery may intensify spiritual experiences, possibly as a coping response to perceived risk. Addressing spiritual needs in perioperative care—particularly among women, older adults, and oncology patients—could enhance holistic care and improve patient well-being.

## 1. Introduction

The concept of patient-centered care was introduced in 2001 by the Institute of Medicine (now the National Academy of Medicine) as one of six core goals for improving healthcare systems. It emphasizes care that respects and responds to individual preferences, needs, and values, ensuring that patient values guide all clinical decisions [1]. This approach is holistic, addressing not only physical, but also psychological, social, and spiritual needs [2].

In contemporary Western medicine, there is growing recognition of the connection between spirituality and health, and the need for healthcare professionals to consider patients’ spiritual beliefs [3,4]. Important medical organizations, including the World Psychiatric Association and the American Medical Association, advocate for the integration of spiritual care into routine practice [4].

Although there is no single definition, spirituality is commonly understood as the human search for meaning, purpose, and connection—with oneself, others, nature, or the sacred [5]. Unlike religiosity, which involves affiliation with organized religion, spirituality is often viewed as a personal, existential experience rooted in values and beliefs [6,7,8,9,10].

Spirituality and religiosity have been shown to support coping in chronic illness [11,12], yet their role in surgical contexts—particularly among patients with tumoral or non-tumoral pathologies—remains underexplored. Patients with tumors frequently face spiritual distress related to fear, despair, and uncertainty [13], and in such cases, spirituality can offer emotional support, promote psychological adjustment, and enhance quality of life throughout the treatment process [14,15]. Evidence suggests that both spirituality and religiosity contribute meaningfully to mental and physical well-being [16,17,18,19].

Currently, there are over 50 scales for assessing spirituality [20], among which the Daily Spiritual Experiences Scale (DSES), developed by Underwood [21], measures everyday spiritual experiences rather than just religious beliefs. The scale is one of the most balanced, practical, and validated tools available. It consists of a 16-item questionnaire that investigates the frequency of daily spiritual experiences, without focusing on specific religious beliefs or practices. These items reflect a general sense of connection with the divine or with a transcendent dimension as it is experienced in everyday situations [22].

Integrating spiritual needs into medical care has been shown to improve emotional well-being, strengthen the doctor–patient relationship, and increase patient satisfaction [23,24]. Spiritual and religious beliefs may also influence healing processes: a positive spiritual orientation is linked to higher quality of life and better coping, while unresolved spiritual distress can contribute to depression, reduced quality of life, and even increased mortality risk [25]. In oncology, higher levels of spirituality are associated with improved psychological outcomes and overall health [26,27,28,29]. However, despite growing awareness of its importance, spiritual care remains under-implemented in routine clinical practice [15,30]. Research shows that fewer than 40% of specialists working with oncology patients consistently address spiritual needs [31,32,33].

Bucovina, located in northeastern Romania (currently Suceava County), is considered one of the most spiritual regions of the country. It is renowned for its 15th–16th century painted monasteries and for preserving strong cultural and religious traditions [34,35]. The region’s spiritual landscape has been shaped by its multiethnic history and successive political transitions, including the Habsburg annexation in 1774, which introduced Catholic influences into a predominantly Eastern Orthodox population [36,37]. Ultimately, the concept of the “homo bucovinensis” emerged as a model of tolerance and cultural coexistence, highlighting the region’s ability to preserve its diverse heritage [38].

Today, the confessional structure reflects this diversity: 76.66% of the population identify as Orthodox and 8.49% as Roman Catholic, with smaller proportions adhering to Protestant and Neo-Protestant denominations [39,40].

This study aims to assess the level of spirituality among residents of Bucovina, comparing patients with a history of surgical interventions to healthy individuals from the same region.

## 2. Material and Methods

The present study is a case-control and cross-sectional study, conducted in the Bucovina region of Romania, located in the northeastern part of the country (currently Suceava County), between March 2023 and April 2024. A convenience sampling method was used to recruit participants, based on their availability and willingness to participate. While this approach limits the generalizability of the findings, it is commonly employed in exploratory studies and allowed for timely data collection.

A total of 102 individuals were recruited and divided into two equal groups of 51 participants each: one group consisting of patients who had undergone a surgical intervention in the past, and a second group of healthy individuals who had not undergone any surgical procedure. All participants from both groups completed the DSES questionnaire [21].

### 2.1. Participant Selection

Participants

This study included two groups of adult participants from the Bucovina region (Suceava County, Romania). All participants provided informed consent prior to inclusion in the study, and the patients voluntarily completed the DSES questionnaire. The DSES questionnaire was administered by members of the research team who had received prior training in standardized data collection procedures. Participants completed the questionnaire either independently or with minimal guidance, depending on their preference and reading ability. For surgical patients, data collection took place in the outpatient clinic of the “Sfantul Ioan cel Nou” County Emergency Clinical Hospital. Healthy participants from the control group completed the questionnaire either at home or in a quiet setting provided by the research team, ensuring privacy and minimal distractions.

Surgical group (n = 51)

Patients with a history of surgical interventions were recruited through convenience sampling from the outpatient clinic of “Sfantul Ioan cel Nou” County Emergency Clinical Hospital in Suceava, the largest healthcare facility in northeastern Romania. Each participant voluntarily completed the Daily Spiritual Experiences Scale (DSES), and demographic data were collected, including age, sex, area of residence (urban or rural), marital status (married, unmarried, divorced, widowed), education level, religious affiliation, and type of past surgical intervention.

Based on the underlying pathology (malignant vs. benign/non-tumoral), patients were classified into the following categories:

- Non-tumoral digestive pathology (*n* = 25): appendectomy (*n* = 15), cholecystectomy (*n* = 4), gastric ulcer surgery (*n* = 4), inguinal hernia surgery (*n* = 2);
- Benign gynecological tumors (*n* = 8): all underwent myomectomy;
- Oncological pathology (*n* = 7): breast cancer (*n* = 5), thyroid cancer (*n* = 1), colon cancer (*n* = 1);
- Benign urological pathology (*n* = 4): all with prostate adenoma;
- Other non-tumoral conditions (*n* = 7): ophthalmological (*n* = 2), cardiac (*n* = 2), orthopedic (*n* = 2), and neurosurgical (*n* = 1).

Control group (n = 51)

The control group consisted of healthy individuals with no history of surgical or chronic medical treatment. Participants were recruited from the general population of the same geographical region. They completed the same DSES questionnaire, and similar demographic variables were collected (age, sex, area of residence).

Inclusion criteria (both groups):

- Age ≥ 18 years;
- Residence in the Bucovina region (Suceava County, Romania);
- For the surgical group: history of at least one surgical intervention;
- For the control group: no history of surgical or chronic medical conditions; self-reported good health.

Exclusion criteria (both groups):

- Age under 18;
- Diagnosed psychiatric or neurological disorders;
- Terminal chronic illnesses;
- Any diagnosed mental health disorder.

### 2.2. Measures

The Daily Spiritual Experiences Scale (DSES) is a 16-item self-report scale developed by Underwood in the mid-1990s through structured qualitative interviews and quantitative testing across diverse socio-cultural, ethnic, and religious groups. The scale, published in 2011 [41], assesses experiences that reflect a connection with a transcendent dimension. These include feelings of awe, receiving compassionate love and offering altruistic love, spiritual connection, and the desire to draw closer to a divine source; wonder; finding strength, guidance, or comfort from a transcendent source; a deep sense of harmony and inner peace; the feeling of being blessed even in difficult times; and a joy that lifts the person above present concerns. The first 15 items are rated on a 6-point Likert scale ranging from 1 (many times a day) to 6 (never or almost never). Item 16, which assesses the general perception of closeness to God, is rated on a 4-point scale (1 = not at all, 4 = as close as possible), and its score is reversed to maintain consistency in the direction of interpretation [41].

The scale has been translated into 40 languages, including Romanian, and has been used in over 400 studies to date [41]. It has been validated through extensive qualitative testing applied to various population groups, which gives it wide applicability across diverse contexts.

Regarding the validation of this scale in Romania, this was conducted in 2022, when a group of researchers from the Medical Psychology Department of a Romanian medical university evaluated the psychometric properties of the Romanian version of the DSES on a sample of 70 patients diagnosed with depression and 160 healthy volunteers. The conclusions of the Romanian study supported the validity and reliability of the Romanian version of the DSES, and the authors encouraged its use in future research on the spirituality of Romanian patients [42].

### 2.3. Ethical Considerations

The study was approved by the Ethics Committee of the Suceava County Emergency Clinical Hospital (Romania) (no. 5/13.01.2023). The objectives of the study were explained to the participants prior to enrollment, and confidentiality was guaranteed. Participants were assured that there were no risks associated with participating in the study and that they could withdraw at any time. Participation in the study was entirely voluntary. All participants provided informed consent to take part in the study.

### 2.4. Statistical Analysis

All statistical analyses were performed using Statistical Package for the Social Sciences (SPSS) software, version 26 for Windows (IBM Corp., Armonk, NY, USA), employing descriptive statistical methods (mean and standard deviation), the Pearson correlation test, and the independent samples *t*-test. The threshold for statistical significance was set at a probability level of *p* < 0.05.

## 3. Results

### 3.1. Demographic Characteristics

Among the 102 participants, 50 were women (49.02%) and 52 were men (50.98%). Age ranged from 18 to 71 years. The surgical patient group (*n* = 51) included 51% women (*n* = 26) and 49% men (*n* = 25), while the healthy control group (*n* = 51) included 53% women (*n* = 27) and 47% men (*n* = 24). Based on age, both groups were divided into three age categories: 18–30 years, 31–70 years, and ≥ 71 years.

### 3.2. General Differences Between Operated and Non-Operated Patients

A comparative analysis was conducted on the mean spirituality scores between operated patients and healthy individuals, stratified by sex and age group. The DSES scores by age and sex, for both operated and non-operated patients, can be observed in Table 1.

Group Comparisons

Operated patients showed significantly higher spirituality scores (M = 66.27, SD = 16.40) compared to non-operated individuals (M = 55.06, SD = 12.81), with *p* < 0.001.

Sex Differences

Sex significantly influenced spirituality levels. Among operated patients, women had significantly higher mean DSES scores (M = 70.73, SD = 13.10) than men (M = 61.64, SD = 18.36), with a *p*-value of 0.047. A similar pattern was observed in the non-operated group, where women scored higher (M = 59.85, SD = 11.34) than men (M = 49.67, SD = 12.41), and the difference was statistically significant (*p* = 0.004).

Age-related Trends

Age-related trends were also identified. In the non-operated group, younger individuals (18–30 years) had the lowest spirituality scores (M = 42.86, SD = 14.31), significantly lower than those in the 31–70 age group (M = 57.00, SD = 11.58), with *p* = 0.005. In contrast, among operated patients, mean DSES scores increased with age (18–30 years: M = 56.67; 31–70 years: M = 67.38; ≥71 years: M = 68.67), but these differences did not reach statistical significance (*p* = 0.312).

**Figure 1:**
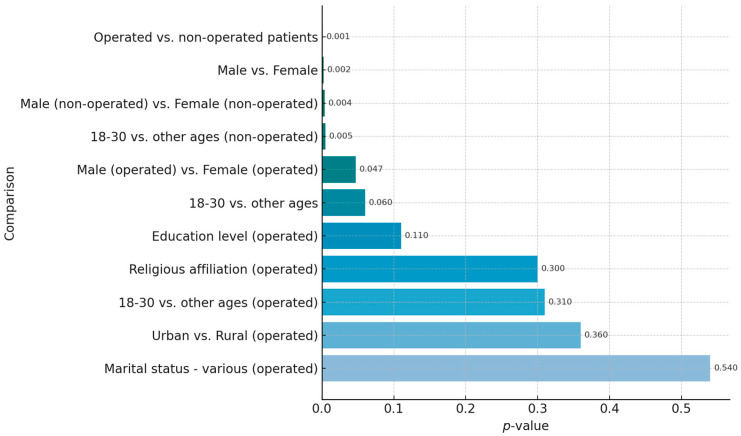
Heatmap of *p*-values for Daily Spiritual Experiences Scale (DSES) score comparisons.

A heatmap of *p*-values was generated to visually summarize the statistical significance of group differences in mean DSES scores across demographic and clinical factors, providing a clear overview of the most significant associations (Figure 1).

These results highlight that both sex and surgical status are significantly associated with spirituality levels, with women and operated patients reporting the highest mean DSES scores. Additionally, younger age appears to be associated with lower levels of spirituality, particularly among non-operated individuals.

### 3.3. Female Gender as a Predictor of Increased Spirituality

**Figure 2:**
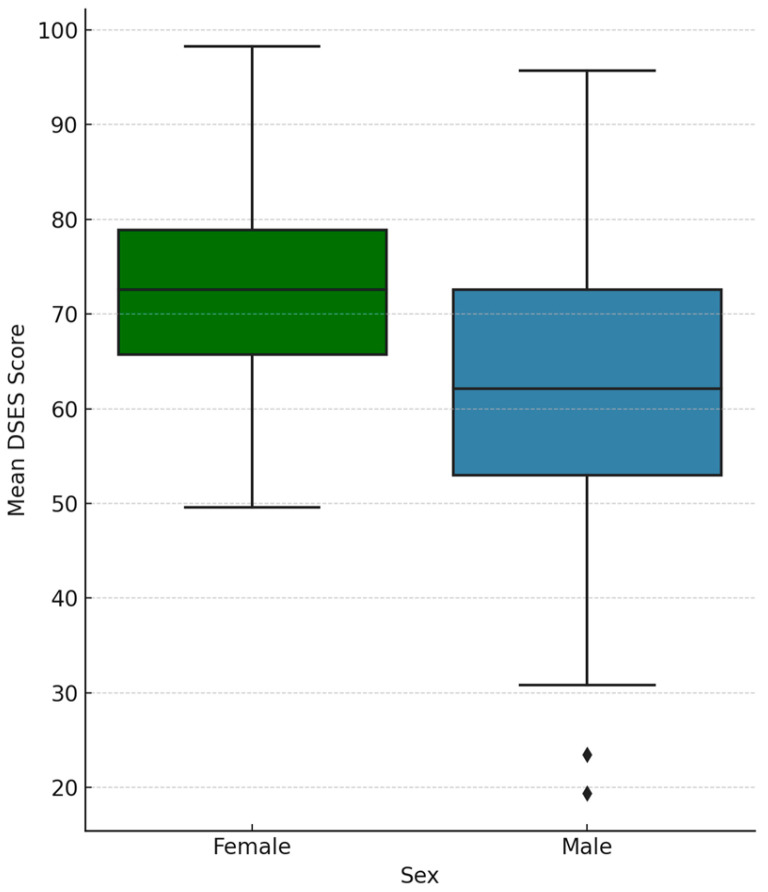
Distribution of Daily Spiritual Experiences Scale (DSES) scores among female and male participants (*n* = 102).

In the entire study sample of 102 participants, women had higher average spirituality scores (M = 65.19, SD = 13.31) compared to men (M = 55.78, SD = 16.70), and this difference was statistically significant (*p* = 0.002). In other words, regardless of surgical history, women reported significantly higher mean DSES scores than men (Figure 2).

**Figure 3:**
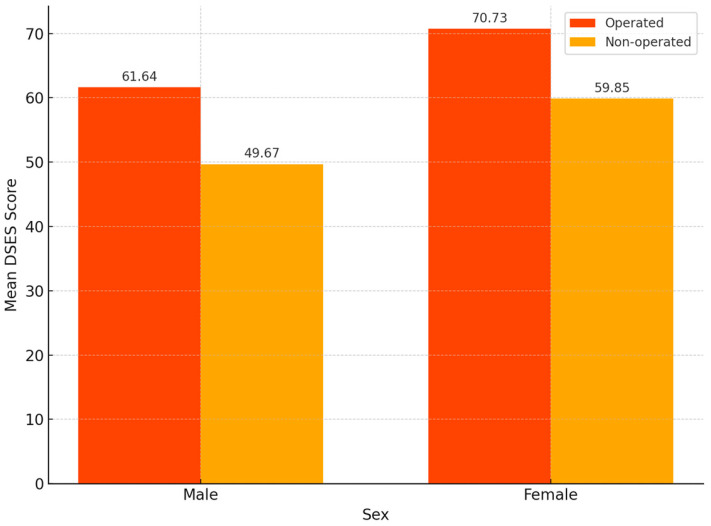
Sex differences in Daily Spiritual Experiences Scale (DSES) scores between operated and non-operated patients (*n* = 102).

Regarding the sub-analysis within the group with surgical interventions, significant gender differences were identified: operated women had a higher mean score (M = 70.73, SD = 13.11) than operated men (M = 61.64, SD = 18.36), with *p* = 0.047 (Figure 3). Similarly, in the non-operated group, significant gender differences were also observed (*p* = 0.004).

### 3.4. Age-Related Trends in Spirituality Levels

Regarding the relationship between age and the mean DSES scores, in both groups we observed that spirituality tends to increase with age, although the differences were not statistically significant at the global level (*p* = 0.060). When analyzing age categories, participants over the age of 70 had the highest mean score (M = 68.67; SD = 20.47), followed by those aged 31–70 (M = 61.88; SD = 13.51), while participants aged 18–30 had the lowest score (M = 49.23; SD = 21.59), showing a trend toward statistical significance (*p* = 0.060).

**Figure 4:**
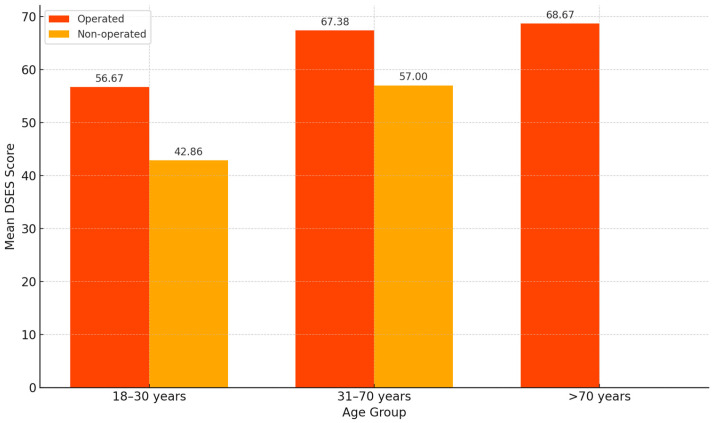
Age-related variation in Daily Spiritual Experiences Scale (DSES) scores by surgical status (*n* = 102).

When comparing mean DSES scores between the two groups by age, we observed that healthy individuals aged 18–30 had a much lower score (42.86) compared to those in the operated group of the same age (56.67) (Figure 4).

### 3.5. Correlation Between Spirituality Scores and Surgical Pathologies

**Figure 5:**
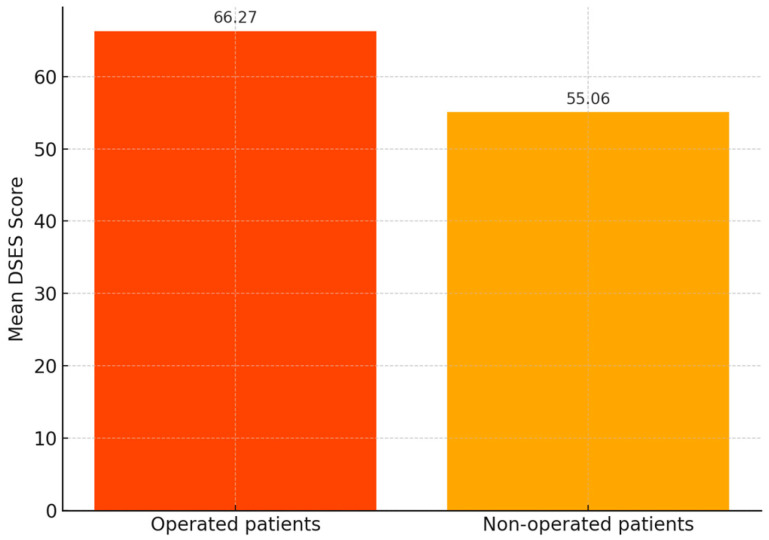
Comparison of mean Daily Spiritual Experiences Scale (DSES) scores between surgically and non-surgically treated patients (*n* = 102).

Patients with a history of surgical intervention recorded a mean DSES score of 66.27 (±16.40), whereas non-operated patients had a significantly lower mean score of 55.06 (±12.81). This difference was statistically significant (*p* < 0.001) (Figure 5).

**Figure 6:**
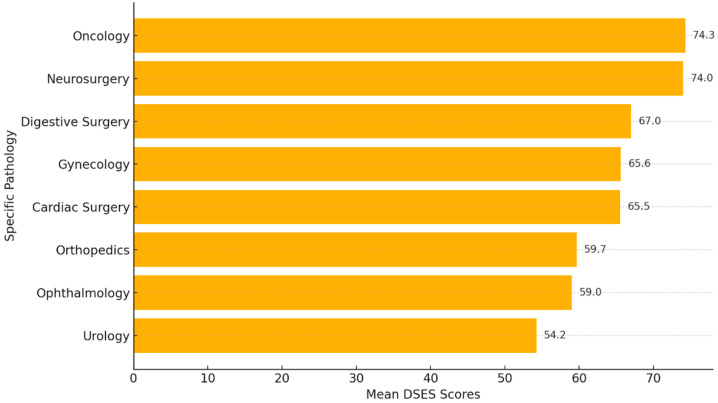
Mean Daily Spiritual Experiences Scale (DSES) scores by specific pathology in surgically treated patients (*n* = 51).

Mean DSES scores were analyzed based on the type of pathology and surgical intervention. Although no statistically significant differences were identified between patients’ mean DSES scores and their specific pathologies, notable variations were observed across groups, with mean DSES scores ranging from 54.2 to 74.3 points (Figure 6).

Patients with a personal history of oncological surgical interventions reported the highest mean spirituality score (M = 74.3), closely followed by those who had undergone neurosurgical procedures (M = 74.0). These values suggest an intensification of spiritual experiences among patients with potentially life-threatening conditions. In contrast, the lowest mean DSES scores were reported by patients with benign urological conditions (M = 54.2), non-tumoral ophthalmological conditions (M = 59.0), and non-tumoral orthopedic conditions (M = 59.7), indicating a lower perceived level of daily spiritual experiences.

### 3.6. Marital Status and DSES Score

In the analysis of the subgroup of operated patients, the following mean DSES scores were observed based on marital status: widowed: M = 70.29; SD = 13.6, married: M = 67.16; SD = 14.08, divorced: M = 66.40; SD = 20.55, and single: M = 58.14; SD = 25.49. Although no statistically significant differences were found between the groups (*p* = 0.538), a trend can be noted: widowed and married individuals tend to report higher average levels of daily spiritual experiences compared to unmarried individuals. This difference may reflect the role of emotional and social support provided by a life partner, or, in the case of widowed individuals, a possible intensification of the spiritual dimension as a coping mechanism in the face of loss. However, due to the small sample sizes for certain categories (e.g., single: *n* = 7; divorced: *n* = 5), these results should be interpreted with caution.

### 3.7. Non-Significant Positive Association Between Education Level and Spirituality

**Figure 7:**
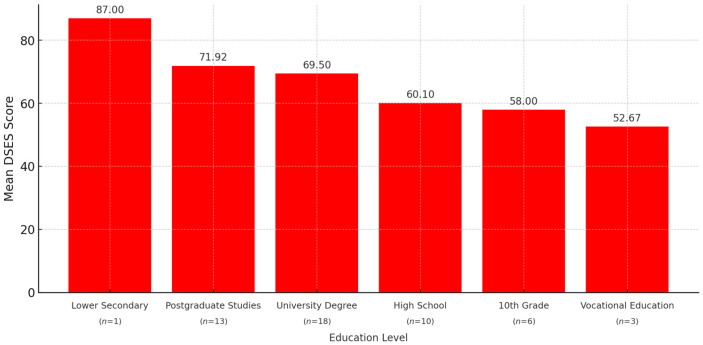
Mean Daily Spiritual Experiences Scale (DSES) scores by educational level in operated patients (*n* = 51).

The analysis of mean DSES scores based on educational level revealed the following means: 4 grades: M = 87.00 (*n* = 1), 10 grades: M = 58.00; SD = 20.18, high school (12 grades): M = 60.10; SD = 19.31, vocational school: M = 52.67; SD = 13.80, higher education: M = 69.50; SD = 14.24, and postgraduate education: M = 71.92; SD = 12.39 (Figure 7).

Although the analysis did not reveal overall statistical significance (*p* = 0.107), a clear trend was observed: mean DSES scores increased with higher levels of education. Individuals with higher and postgraduate education reported the highest levels of daily spiritual experience, while the lowest scores were recorded among those with secondary or vocational education. This trend may suggest that a higher educational level is associated with greater awareness or openness to the spiritual dimension, possibly due to access to more resources for personal reflection, inner development, and cultural diversity.

### 3.8. Religious Affiliation and Level of Spirituality

In the control group, among the 51 participants, 46 identified as Orthodox, with a mean spirituality score of 65.38; one Catholic participant had a score of 63.00; and four Protestant participants recorded the highest mean score of 78.5. Although statistical analyses did not reveal a significant difference (*p* = 0.302), it is noteworthy that Protestant participants reported the highest mean DSES score, suggesting a more intense experience of daily spirituality within this group. Orthodox participants, who constituted the majority of the sample (*n* = 46), had an intermediate mean score, while the single Catholic participant (*n* = 1) had a score similar to the Orthodox average. These results should be interpreted with caution due to the small sample sizes in the Catholic and Protestant categories.

## 4. Discussion

The first study to evaluate the psychometric properties of the DSES in the Romanian population was conducted in 2022 by Popescu et al., who validated the scale for use in Romania [42]. The range of mean item scores in the Romanian version of the DSES was comparable to that of the original English version [22], as well as to the findings of other international studies [43,44]. Moreover, the authors noted that the Romanian version of the DSES effectively captures the spirituality specific to Orthodox individuals, as 78.6% of participants in the case group and 75.9% in the control group identified as Orthodox [42]. This suggests that the scale is culturally appropriate for the Romanian religious context, which is predominantly Orthodox.

To the best of our knowledge, this is the first study to evaluate spirituality scores using the DSES questionnaire in patients who have undergone surgical interventions, and also the first study to assess spirituality among Romanian surgical patients. We consider the present study to be valuable as it demonstrated that Romanian patients with a history of surgery had significantly higher spirituality scores compared to healthy individuals. This finding suggests a heightened vulnerability in the face of surgical procedures and highlights the need for increased attention from healthcare professionals to the spiritual needs of these patients.

Spirituality and religion represent important aspects for many individuals in the general population. According to a recent national survey conducted by the Pew Research Center, approximately 80% of Americans identify with a religion, and more than half (53%) consider religion to play a central role in their lives [45]. Various studies have shown that spiritual beliefs and religious practices are associated with improved mental health, providing patients with hope, optimism, and meaning—all of which are crucial during the perioperative period [46,47].

In the case of Romania, spirituality plays a significant role in the lives of Romanian patients. Population-based studies indicate that it constitutes an integral part of mental well-being and rehabilitation processes [48,49,50].

Surgical interventions, particularly due to their potential risks, can lead to an increase in patients’ religious and spiritual engagement. These aspects have been observed in patients undergoing coronary artery bypass surgery, where religious thoughts provided comfort and reassurance [51]. The fear of surgery and possible complications may prompt patients to seek strength in their faith, with some studies suggesting that religious practices can help patients manage anxiety [52].

In our study, we observed that patients who underwent surgery, whether for tumoral or non-tumoral conditions, subsequently reported significantly higher mean DSES scores, with statistically significant differences. Among all patients, those with oncological conditions exhibited the highest mean DSES scores. This finding suggests that patients who have undergone surgery may experience a deeper engagement with the spiritual dimension of daily life. It is possible that the experience of a surgical intervention—often perceived as a major and sometimes life-threatening event—leads to a re-evaluation of personal values, an increased sense of vulnerability, and a greater openness to spiritual reflection.

Studies have shown that surgical interventions, especially those involving life-threatening conditions such as cancer, confront patients with their own mortality, leading to increased spiritual and religious engagement as a means of coping with existential concerns [53,54]. In the case of individuals diagnosed with cancer, some research suggests that patients may wish for the medical team to acknowledge and address their spiritual or religious needs [55,56]. However, there is a lack of in-depth studies exploring how this dimension is actually addressed by healthcare professionals or what patients’ preferences are regarding the assessment of spiritual well-being in the context of illness.

In our study, women reported daily spiritual experiences more frequently than men, consistent with findings from other studies and cultural contexts [44,57,58]. Galek et al. reported that women have a greater need for belonging, meaning, hope, beauty, and acceptance of death—aspects related to spiritual experience. These needs are more commonly expressed by women than by men, suggesting that women may engage more deeply in spirituality to fulfill these needs [59].

Regarding the particularity of spirituality among Romanian women, Romanian-born anthropologist Iulia Hasdeu observed that, traditionally, women are more actively involved in religious and spiritual practices, often assuming roles that emphasize nurturing and community-building. This involvement may lead to more frequent spiritual experiences as an integral part of their daily lives [60]. Women may be more inclined to seek spiritual experiences as a source of comfort and strength, especially when facing health challenges or emotional stress. For example, McCauley et al. reported that women with chronic conditions such as arthritis experienced more frequent spiritual experiences, which were associated with improved mental health [61]. Other authors have also shown that spiritual experiences contribute to greater self-esteem, a sense of purpose, and psychological well-being in women, who appear to derive more psychological benefits from spirituality, thereby reinforcing their spiritual practices [62]. Interestingly, some studies have suggested that the gender difference in spirituality scores may diminish with age, as both men and women tend to exhibit similar levels of spiritual engagement in later life [63].

Studies have shown that as people age, they often engage in life review, reflecting on past experiences and seeking meaning in their lives. This process may lead to a greater emphasis on spirituality as a source of meaning and purpose [64,65]. In this context, awareness of mortality becomes more prominent in later life, prompting individuals to seek comfort and understanding through spiritual beliefs and practices [66,67]. The theory of gerotranscendence suggests that aging is accompanied by a shift toward a more transcendent perspective, in which individuals become less focused on material concerns and more engaged with spiritual and existential questions [67]. These factors may help explain the findings of our study, in which we observed that spirituality scores increase with age in both men and women.

Various authors have shown that spirituality often deepens following a period of bereavement after the loss of a life partner. In this regard, Richards et al. demonstrated that individuals who lost their partners to AIDS developed a deeper sense of spirituality, with 77% reporting an increase in spiritual beliefs and practices [68]. Furthermore, grief-related life review therapy has been found to enhance spiritual well-being and reduce depression among widowed individuals [69,70]. Research on post-traumatic spiritual growth in women who have lost their husbands has shown that spiritual change is a significant aspect of personal growth following bereavement [71]. However, despite the known benefits of spirituality during and after the grieving process, many healthcare professionals lack the necessary training to effectively address the spiritual needs of their patients [72].

Spiritual care is a fundamental component of holistic healthcare, aiming to address the physical, psychological, social, and spiritual dimensions of patient care. Nurses, who spend significant time with patients, are ideally positioned to provide this type of care; however, they often lack the necessary training and guidance [73]. Nursing education programs are increasingly incorporating spiritual care to enhance students’ competencies in this area. These programs aim to strengthen nurses’ ability to deliver integrative and responsible spiritual care, which is essential for patient recovery and the achievement of health goals [74,75]. Integrating spiritual assessments into clinical practice, through the use of validated tools such as the DSES, represents an essential step toward a holistic approach to patient care. By identifying and understanding the spiritual dimensions of human experience, healthcare professionals can more effectively address the emotional, existential, and spiritual needs of patients. This approach not only fosters a more empathetic therapeutic relationship but may also contribute to improved overall well-being and quality of life, particularly in the context of chronic illness or profound suffering.

This study also has several limitations that should be acknowledged. First, the control group was selected using a convenience sampling method, which, although practical, may introduce selection bias and limit the representativeness of the sample. Second, the sample size of surgically treated patients was relatively small, and some subgroups (e.g., based on specific pathologies or marital status) were underrepresented. As a result, the absence of statistically significant differences between surgical pathology types should be interpreted with caution, as it may be due to limited statistical power rather than a true lack of effect. Furthermore, the study was conducted in a region characterized by cultural and religious homogeneity, with the vast majority of participants identifying as Christian Orthodox. While this reflects the local population structure, it may have influenced the distribution of DSES scores, as shared religious values and practices could lead to similar patterns of spiritual expression. Therefore, the findings may not be fully generalizable to populations with different religious or cultural backgrounds, such as those from more diverse urban areas or non-Christian communities.

## 5. Conclusions

According to the findings of this study, the majority of surgical patients reported a high level of daily spiritual experience, significantly greater than that of healthy adults in the control group. Although this level of spirituality does not appear to depend directly on the type of surgical pathology, it may still be influenced by it. Additionally, female sex and older age were consistently associated with higher spirituality scores.

The elevated level of spirituality observed among surgical patients may reflect a greater need for emotional and spiritual support during the perioperative period. In this context, the integration of standardized tools into clinical assessments could help healthcare providers identify and address patients’ spiritual needs more effectively. This would support the delivery of personalized, patient-centered, and holistic care, particularly in vulnerable populations such as oncology patients, older adults, or those facing high-stress procedures.

Furthermore, these preliminary data highlight the need for expanding research at the national level to assess daily spiritual experiences across various regions of Romania. Comparing results between different geographical and cultural areas could uncover important socio-cultural, religious, and spiritual variations. These insights could inform public health policies and institutional guidelines aimed at integrating spiritual care into healthcare systems—both at the hospital level and within community-based health services.

Ultimately, recognizing and systematically addressing the spiritual dimension of health may not only improve patient satisfaction and psychological resilience, but also contribute to the overall effectiveness and humanization of healthcare services.

## Figures and Tables

**Table 1:** Comparison of mean spirituality scores by age and gender between operated and non-operated patients.

Variable Subgroup		Operated Patients		Non-Operated Patients		*p*-Value
		N	(Mean ± SD)	N	(N, Mean ± SD)	
Gender	Female	26	70.73 ± 13.1	27	59.85 ± 11.34	0.004 **
	Male	25	61.64 ± 18.36	24	49.67 ± 12.41	0.047 *
Age group (years)	18–30	6	56.67 ± 27.38	7	42.86 ± 14.31	0.005 **
	31–70	39	67.38 ± 13.54	44	57.00 ± 11.58	—
	≥71	6	68.67 ± 20.47	-	—	—

SD = standard deviation; statistical significance: *p* < 0.05 (*), *p* < 0.01 (**).

## Data Availability

The original contributions presented in this study are included in the article. Further inquiries can be directed to the corresponding author.
